# Simultaneous observation of particle and wave behaviors of entangled photons

**DOI:** 10.1038/srep42539

**Published:** 2017-02-13

**Authors:** Zhong-Xiao Man, Yun-Jie Xia, Nguyen Ba An

**Affiliations:** 1 Shandong Provincial Key Laboratory of Laser Polarization and Information Technology, Department of Physics, Qufu Normal University, Qufu 273165, China; 2 Center for Theoretical Physics, Institute of Physics, Vietnam Academy of Science and Technology (VAST), 18 Hoang Quoc Viet, Cau Giay, Hanoi, Vietnam

## Abstract

We theoretically study wave-particle duality of two entangled photons in the spirit of quantum version of delayed-choice experiments using Hadamard gate controlled by the quantum state of an ancilla and show that the two photons may globally exhibit particle-like, wave-like or simultaneously both particle-like and wave-like behavior. We prove that the obtained results cannot be satisfactorily explained by any hidden-variable theory. We also propose an efficient and experimentally feasible scheme without using any ancilla and controlled-gates to directly (i.e., without postselection) observe the two-photon wave-particle superposed state as well as the continuous transition of their behavior between wave-like one and particle-like one.

Wave-particle duality is the most fundamental description of the nature of quantum objects which behave as classical particles in one measurement apparatus, but as classical waves in another measurement apparatus. This is very well demonstrated in the so-called delayed-choice experiments^1^,^2^,^3^,^4^,^5^,^6^. In the single-photon delayed-choice experiment, a photon is sent into a Mach-Zehnder interferometer (MZI) and split by a balanced beam splitter (BBS) into two traveling paths. After the photon has entered the MZI, one randomly determines whether to recombine the two paths or not by a second BBS. If the second BBS is absent, each detector behind the two paths fires with an equal probability of 1/2, revealing particle property of the photon. By inserting the second BBS, the which-way information of the photon is erased so that the photon exhibits wave property reflected via the dependence of detection probability of each detector upon the phase difference between the two routes. The results of such delayed-choice experiments rule out the possibility of the photon being able to ‘know’ beforehand the specific experimental setting. It also proves that the erasure of the path information is necessary for the photon to form interference between the two paths. In refs 7 and 8, Scully and collaborators proposed the so-called quantum eraser in an entangled atom-photon system. If the path information of the photon (atom) is exposed by the atom (photon), one cannot observe the interference patterns of the photon (atom). In order to recover the interference patterns, one should erase the which-way information^7^,^8^. The quantum erasure schemes under various contexts were realized in refs 9, 10, 11, 12, 13, 14, 15.

In addition to the single-quantum situation, the interferences in the case of multiphoton entangled states are also extensively studied, which can generally be classified into two types: the first type is to show superresolution effects by manipulating all the photons together in a single interferometer^16^,^17^,^18^,^19^,^20^,^21^,^22^,^23^,^24^, while the second type is to reveal genuine multiphoton interference by sending each photon into an independent interferometer^25^,^26^. Spatial quantum interference of two-mode entangled three-photon state has been observed experimentally in a single interferometer, where the interference fringes are three times denser in comparison to the single-photon state^19^,^23^. By contrast, in Ref. 26, three photons that are prepared in Greenberger-Horne-Zeilinger entangled state^27^ are sent into three independent MZIs, demonstrating three-photon interference that does not originate from two-photon nor single photon interference. To analyze the wave-particle duality for multiphoton states, higher-order duality relations are also proposed^28^,^29^.

In a recent development, a quantum version of delayed-choice schemes has been proposed^30^ where the presence of the second BBS is controlled by a quantum ancilla. The second BBS is removed (inserted) if the ancilla is in the state |0〉 (|1〉). Because the ancilla is quantum it can be prepared in a superposed state of |0〉 and |1〉 and made entangled with the test system through controlled operation. Then the test system can be projected onto a superposition of the wave-like and particle-like states depending on the outcome of a suitable measurement of the ancilla. That is, the wave-like and particle-like behaviors of the test system can be observed at the same time in one and the same experimental setting. Through varying the superposition coefficients of |0〉 and |1〉 of the ancilla, the test system experiences a continuous morphing between the particle-like and wave-like behaviors. This quantum delayed-choice scheme has been successfully demonstrated in the context of photons^31^,^32^,^33^,^34^ and other systems^35^,^36^,^37^,^38^. In the existing implementation of the quantum delayed-choice experiment^30^, one should employ a quantum ancilla, perform controlling operation on the ancilla and the test system, and make proper measurement on the ancilla.

Although the particle-like and wave-like behaviors of a single photon has been observed simultaneously in one and the same experimental setup, the possibility of such observation regarding multi-photon states remains to be explored. In the present work, we consider the quantum version of delayed-choice experiments designed so that to simultaneously observe both particle and wave behaviors of two entangled photons. It is found that even though each individual photon independently behaves as a particle, their collective properties may not only be particle-like or wave-like but also be both wave-like and particle-like at the same time. We particularly demonstrate the continuous transition between wave and particle behaviors for the entangled photons. We also provide a proof that no hidden-variable (HV) theory can satisfactorily explain the obtained results. Furthermore, we propose an efficient and experimentally feasible scheme which replaces the ancilla, the controlled-Hadamard operation by only a polarizer and a polarization beam splitter (PBS). Our scheme is simple and easily implemented by current technologies because it avoids using controlled-Hadamard gate whose construction and execution are technically difficult. Importantly, in our scheme the wave-particle superposed state of the two photons can be observed directly at the output of the device without any postselection since no measurements at all are required.

## Results

### Quantum ancilla based scheme

**Figure 1:**
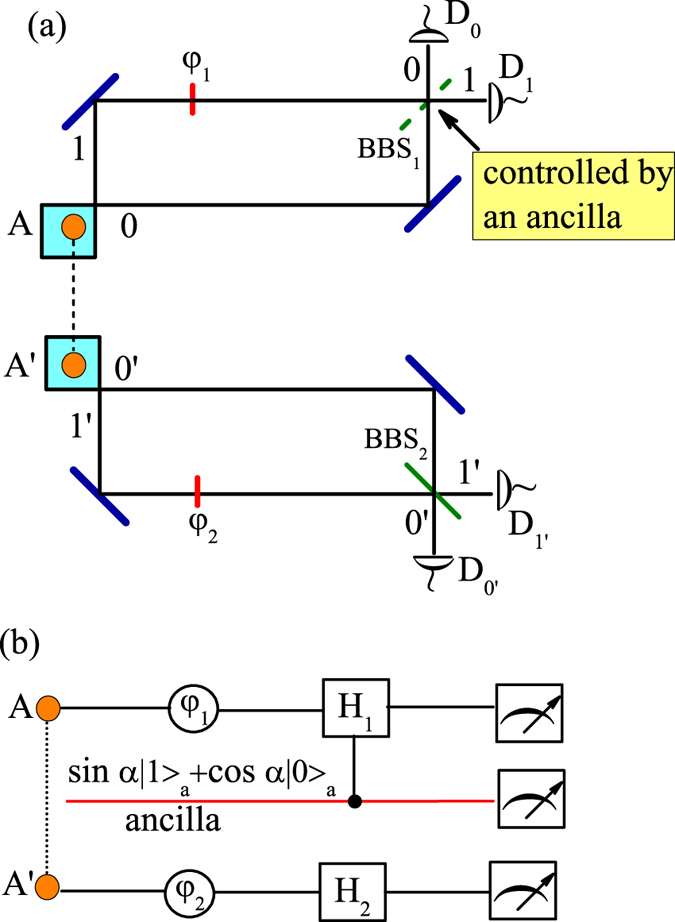
(**a**) Two entangled photons *A* and *A*′ are input into two independent interferometers and are transformed to the entanglement of the four paths 0, 1, 0′ and 1′, as given in Eq. (1). * φ*_1_ and *φ*_2_ are phase shifters. The existence of BBS_1_ is controlled by the state of an ancilla, while BBS_2_ is always inserted. (**b**) The equivalent quantum network. The BBSs correspond to Hadamard gates. The ancilla is prepared in an arbitrary state, |*ψ*〉_*a*_ = sin*α*|1〉_*a*_ + cos*α*|0〉_*a*_ and applied to control the operation of H_1_.

We consider a device consisting of two independent interferometers as shown in Fig. 1(a) to implement the delayed-choice of two-photon interference. At the input sites of the device, two photons *A* and *A*′ entangled in certain degrees of freedom are sent into the top and the bottom interferometers, respectively, and their states are transformed to the path-entanglement of the interferometers being of the form


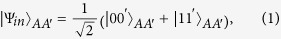


where |*n*〉 (with *n* = 0, 0′, 1, 1′) represents that there is a photon in the path *n*. In the path 1 (1′), *φ*_1_ (*φ*_2_) denotes the phase shifter introducing a phase difference *φ*_1_ (*φ*_2_) for the paths 0 and 1 (0′ and 1′). At the output sites, the two paths 0′ and 1′ of the bottom interferometer are always combined by the BBS_2_ (i.e., the bottom interferometer is always closed). As for the paths 0 and 1 of the top interferometer, whether to combine them by the BBS_1_ or not (i.e., the top interferometer is either closed or open) is controlled by the state of an ancilla. In Fig. 1(b), we sketch an equivalent quantum network to describe the experiment, where Hadamard gates H_1_ and H_2_ play the roles of BBS_1_ and BBS_2_, respectively. Whether the H_1_ operates or not for the photon *A* depends upon the ancilla being in the state |1〉_*a*_ or |0〉_*a*_. The photons are detected simultaneously in the form of two-photon coincidence and the collective detection probabilities are employed to characterize the global characters of the two photons as wave-like or particle-like. In the case when the BBS_1_ exists (that is, the ancilla is in the state |1〉_*a*_), the output state of the two photons takes the form


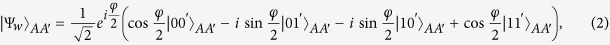


with *φ* = *φ*_1_ + *φ*_2_. From Eq. (2), one can see that the collective detection probabilities are related to the phase *φ*, implying that the two photons exhibit interference. Therefore their global behavior is wave-like and the state (2) is taken for the wave state of the two photons (indicated by a subscript *w*). By contrast, if the ancilla is in the state |0〉_*a*_ and the BBS_1_ is removed, the output state of the two photons turns out to be


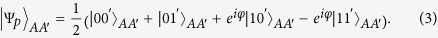


Evidently, all the possible collective detection probabilities of the two photons are equal to 1/4, independent to the phase *φ*, which implies that the two photons exhibit particle-like behavior. Hence, we take the state (3) to be the particle state of the two photons (indicated by a subscript *p*).

To simultaneously observe the wave and particle aspects of the two photons, we prepare the ancilla in a superposed state |Ψ〉_*a*_ = sin*α*|1〉_*a*_ + cos*α*|0〉_*a*_, which formally implies that a quantum beam splitter is applied. In this case, the output state of the two photons are entangled with the ancilla as


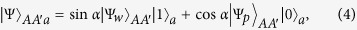


with |Ψ_*w*_〉_*AA′*_ and |Ψ_*p*_〉_*AA′*_ being the wave and particle states of the two photons defined in (2) and (3), respectively. The two-photon state can be post-selected via a measurement on the ancilla. For example, if the ancilla is measured in the basis {|+〉_*a*_, |−〉_*a*_} 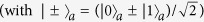, the state of the photons will be projected onto a wave-particle superposed state |Ψ_*wp*_〉_*AA′*_ = cos*α*|Ψ_*p*_〉_*AA′*_ ± sin*α*|Ψ_*w*_〉_*AA′*_ conditioned on the measurement outcomes |+〉_*a*_ or |−〉_*a*_ of the ancilla which happens with an equal probability of 1/2.

In the following, we shall prove that no HV theory, in which particle and wave are realistic properties of two entangled photons, can explain this delayed-choice experiment. Following the conditions for a single photon^30^, a satisfactory HV model should reproduce the quantum mechanical statistics and for a given entangled pair of photons the global behavior of being particle-like or wave-like is intrinsic, i.e., does not change during their lifetime. In the basis |*A*〉⊕|*A′*〉⊕|*a*〉 = {|00′0〉, |00′1〉, |01′0〉, |01′1〉, |10′0〉, |10′1〉, |11′0〉, |11′1〉} the statistics for the joint measurements of the two photons and the ancilla in the state (4) is


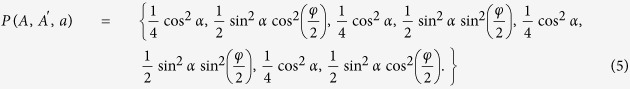


We shall reveal a contradiction of the HV model between the reproduction of the statistics *P(A, A*′, *a*) and keeping consistent behaviors of wave and particle during the experimental process.

*Proof*. Suppose there is an extra degree of freedom *λ*, which is the hidden variable and determines particle-like (*λ* = *p*) or wave-like (*λ* = *w*) behavior of the two entangled photons. According to the standard conditions for probability distributions, we have


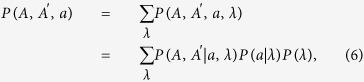


where *P(A, A*′|*a, λ*) is the collective probability of detecting *A* and *A*′ conditioned on the given values of *a* and *λ, P(a*|*λ*) is the probability of *a* (being 0 or 1) conditioned on the value of *λ*, and *P(λ*) is the probability of the source to emit the wave-like or particle-like photons. If the source emits two entangled photons with the wave-like property and meanwhile the BBS_1_ is inserted (*a* = 1), then the collective statistics is consistent with our previous derivation of the wave state of the two photons, namely,


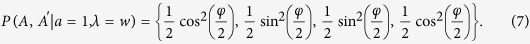


Similarly, if the emitted entangled photons possess particle-like property and meanwhile BBS_1_ is absent (*a* = 0), then the collective statistics read


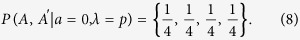


In case the wave-like photons (*λ *= *w*) are injected into the device in the absence of BBS_1_ (*a *= 0), whereas the particle-like photons (*λ *= *p*) are input in the presence of BBS_1_ (*a = *1), the collective statistics are expressed respectively as


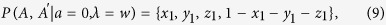


and


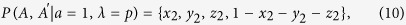


in which the distributions *x*_*i*_, *y*_*i*_, *z*_*i*_ (*i *= 1, 2) are unknown. We assume that the source randomly emits particle-like or wave-like entangled photons with probability *f* or 1 − *f*, respectively, namely, *P(λ*) = {*f*, 1 − *f*}. The conditional probability distributions of the ancilla *a* and the hidden variable *λ* are supposed as


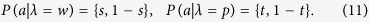


From Eqs (5) and (6) with the constraints (7) and (8) we obtain


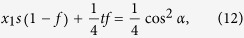



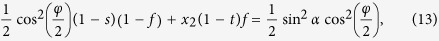



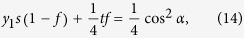



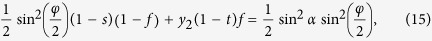



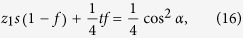



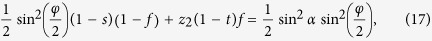



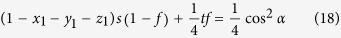



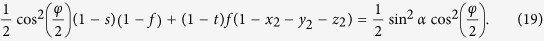


The above Eqs (12),(13),(14),(15),(16),(17),(18),(19), contain three types of solutions. The first type is *x*_1_ = *y*_1_ = *z*_1_ = 1/4, which implies that wave-like photons in the device without BBS_1_ have particle-like statistics: *P(A, A*′|*a* = 0, *λ* = *w*) = {1/4, 1/4, 1/4, 1/4}. The second type solution is *x*_2_ = cos^2^(*φ*/2)/2, *y*_2_ = sin^2^(*φ*/2)/2, *z*_2_ = sin^2^(*φ*/2)/2 which indicates that particle-like photons in the device with BBS_1_ have wave-like statistics: *P(A, A*′|*a* = 1, *λ* = *p*) = {cos^2^(*φ*/2)/2, sin^2^(*φ*/2)/2, sin^2^(*φ*/2)/2, cos^2^(*φ*/2)/2}. These solutions are not acceptable, as the photons show incompatible behaviors. The last type solution is *s *= 0, *t *= 1 and *f *= cos^2^*α*, which means that the randomly emitted particle-like and wave-like photons have a distribution *P(λ*) = {cos^2^*α*, sin^2^*α*} which is identical to the probability distribution of the ancilla *P(a*) = {cos^2^*α*, sin^2^*α*}. Moreover, whenever the source emits particle-like photons the value of *a* is found to be 0, that is *P(a*|*λ *= *p*) = {1, 0} and the BBS_1_ is absent. On the other hand, when it emits wave-like photons the value of *a* is measured as 1, that is *P(a*|*λ *= *w*) = {0, 1}, so the BBS_1_ is inserted. The hidden variable *λ* and the ancilla *a* are perfectly correlated. Although the probability distribution *P(λ*) is identical to *P(a*), namely, the hidden variable completely determines the values of *a*, the probability distribution *P(a*), given by the parameter *α* of the ancilla’s state, is subjectively determined by the experimenter’s preparation. As was verified in ref. 30, if the hidden variable *λ* completely determines *a*, then *λ* itself cannot be determined by the setting *α* in preparing *a*. Therefore, we conclude that the HV model that could reproduce the statistics *P(A, A*′, *a*) cannot keep consistent behaviors of wave and particle of the entangled photons.

### Proposed experimentally feasible scheme

**Figure 2:**
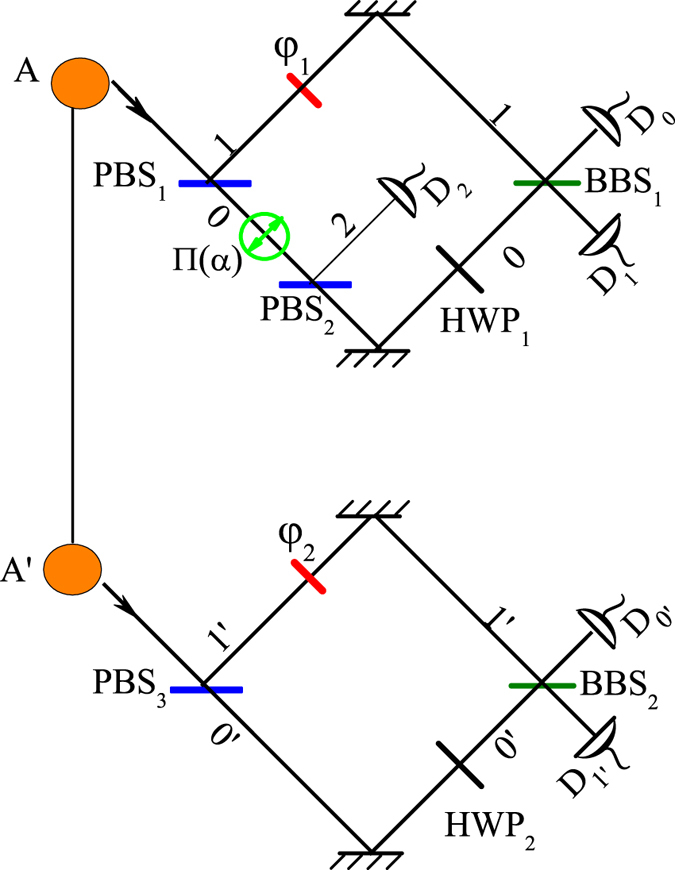
An experimental scheme for demonstrating both wave and particle behaviors of two photons *A* and *A*′ initially being in polarization-entanglement. After the PBS_1_ and PBS_3_, the polarization-entanglement of the photons is transformed to path-entanglement. *φ*_1_ and *φ*_2_ are the phase shifters. Π(*α*) is the polarizer inserted in path 0 where an additional PBS, i.e., PBS_2_, and the corresponding detectors D_2_ are also placed. HWP_1_ and HWP_2_ denote the half-wave plates. The paths 0, 1 and 0′, 1′ are combined by BBS_1_ and BBS_2_, respectively.

In this section, we propose an experimentally feasible scheme to implement the simultaneous observation of both aspects of wave and particle of two entangled photons. Our scheme is shown in Fig. 2, where two photons *A* and *A*′ are prepared initially in the polarization entanglement state as


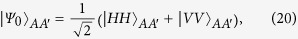


where |*H*〉 and |*V*〉 represent the photon being in the horizontal and vertical polarizations, respectively. In order to transform the polarization entanglement to the path entanglement, two PBSs, PBS_1_ and PBS_3_, are placed at the input sites of the device. Since the PBS transmits (reflects) the photon with horizontal (vertical) polarization, after PBS_1_ and PBS_3_ the state (20) is transformed to


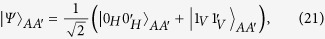


where |*n*_*H(V*)_〉 means that there is a horizontally (vertically) polarized photon in the path *n *= {0, 0′, 1, 1′}. In paths 1 and 1′, we use the phase shifters denoted by *φ*_1_ and *φ*_2_ to vary the phase differences of the two photon paths. In the path 0, we use a polarizer Π(*α*) to transform the photon state |0_*H*_〉 to sinα|0_*H*_〉 + cosα|0_*V*_〉. After the polarizer, another PBS_2_ is placed in the path 0. Therefore, the photon in the path 0 with the vertical polarization is reflected by PBS_2_ and detected at the detector D_2_, otherwise it is transmitted through the PBS_2_, which means that there is nothing in this path. To ensure the possible interference, we use two half-wave plates (HWPs), HWP_1_ and HWP_2_, to rotate the horizontal polarizations of the photons to the vertical polarizations. In the end of the device, the two photon paths 0 and 1 (0′ and 1′) are combined by BBS_1_ (BBS_2_).

Remarkably, instead of using a real quantum ancilla to control the existence of BBS_1_, in our scheme the polarizer Π(*α*) and PBS_2_ play the role of controller. If we set *α* = 0, the photon in path 0 is vertically polarized and the PBS_2_ is equivalent to a mirror. In this case, the total device is equivalent to the scheme described in Fig. 1 with the top interferometer being open, i.e., the BBS_1_ being removed, so that the two photons exhibit particle-like behaviors. In contrast, if we set *α *= *π*/2, the photon in path 0 is horizontally polarized and the PBS_2_ is equivalent to nothing. The total device is just the scheme described in Fig. 1 with two closed interferometers so that the two photons are wave-like. Of interest is the setting 0 < *α *< *π*/2, under which the PBS_2_ is semitransparent, being equivalent to a superposition of existence and absence of the BBS_1_ for the scheme described in Fig. 1. Therefore, with 0 < *α *< *π*/2 we obtain a superposed state of wave and particle of the two photons.

Our calculations yield the output state of the two photons as


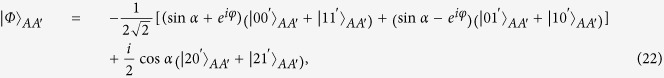


in which we have omitted the polarization indices and taken into account the action of the mirror as |*n*〉 → *i*|*n*〉. As can be derived from Eq. (22), the collective detection probabilities of the two photons are


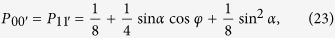



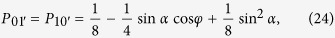



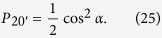


**Figure 3:**
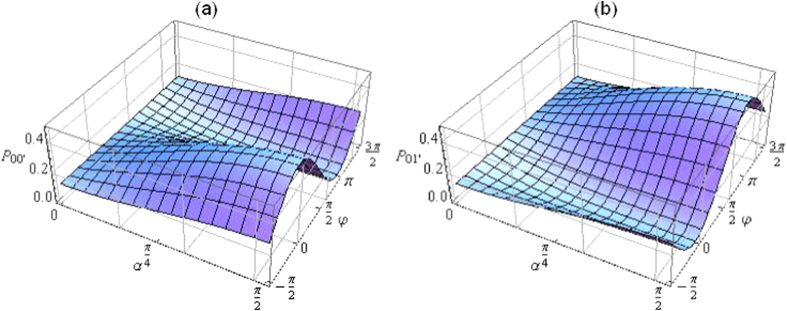
The collective detection probabilities (**a**) *P*_00′_ = *P*_11′_ and (**b**) *P*_01′_ = *P*_10′_, Eqs (23) and (24), of the two photons as functions of *α* and *φ*. The morphing between the particle-like (*α *= 0) and the wave-like (*α *= *π*/2) behavior of the two photons can be seen by changing *α* from 0 to *π*/2 or vice versa.

By setting *α* = 0 in Eqs (23),(24),(25), we get *P*_00′_ = *P*_11′_ = *P*_01′_ = *P*_10′_ = 1/8 and *P*_20′_ = 1/2, all of which are independent to *φ* reflecting the particle-like character of the two entangled photons. By setting *α *= *π*/2, we have nonzero collective probabilities *P*_00′_ = *P*_11′_ = cos^2^(*φ*/2)/2 and *P*_01′_ = *P*_10′_ = sin^2^(*φ*/2)/2, which are related to *φ* reflecting the wave-like character of the two entangled photons. For 0 < *α *< *π*/2, the two photons exhibit at the same time both the wave-like and particle-like characters, which is most evident when *D*_2_ does not respond, in which case the state (22) collapses into


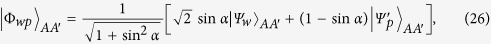


where |Ψ_*w*_〉_*AA′*_ is defined in (2) as a wave state and 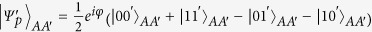 is a particle state (i.e., the detection probabilities are *φ*-independent) as that defined in (3) up to a unitary operation 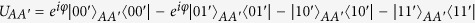 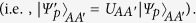 Therefore, the state (26) is a superposition of the wave-like state and the particle-like state (indicated by a subscript *wp*). The dependences of *P*_00′_ = *P*_11′_ and *P*_01′_ = *P*_10′_ on *α* and *φ* are displayed in Fig. 3, reflecting clearly the particle-like (wave-like) behavior at *α *= 0 (*α *= *π*/2) and a continuous transition between these two extreme cases as *α* gradually varies from 0 to *π*/2 or vice versa.

In the following, we analyze how possible experimental imperfections in the state generation and detection processes affect our results. Due to practical device/operation imperfections the desired maximally entangled state |Ψ_0_〉_*AA′*_ may not be generated. Instead, a non-maximally entangled state 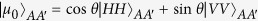 or, more generally, a classical mixture as a Werner-like state^39^


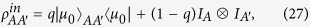


with 0 ≤ *q* ≤ 1 and 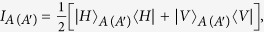 is obtained. In this case, the output state of the two photons can be derived as


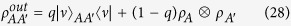


where


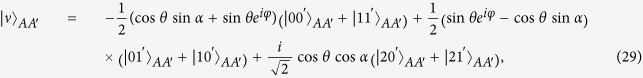



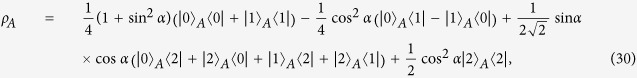


and


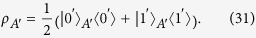


**Figure 4:**
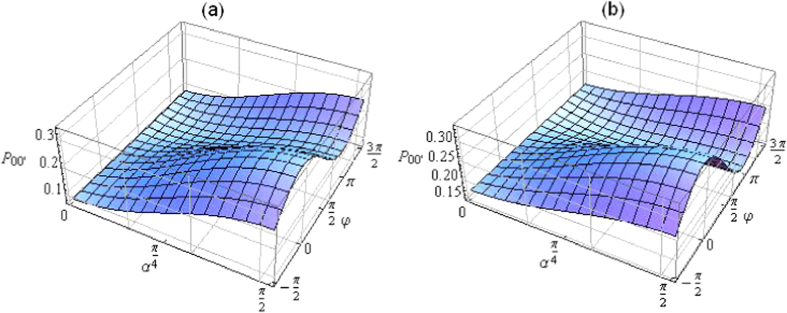
The collective detection probabilities *P*_00′_ = *P*_11′_ of the two photons as functions of *α* and *φ* for the Werner-like input state (27) for *q *= 1/2 and for (**a**) *θ *= *π*/8 and (**b**) *θ *= 3*π*/8. The morphing between the particle-like (*α *= 0) and the wave-like (*α *= *π*/2) behavior of the two photons can still be seen by changing *α* from 0 to *π*/2 or vice versa.

Clearly, the above state (28) is reduced to Eq. (22) when *q *= 1 and *θ *= *π*/4 corresponding to the maximally entangled input state |Ψ_0_〉_*AA′*_. Note that for *q *= 0 the input state of the two photons is separable (here each photon is in a maximally mixed state) and no interference patterns appear at all (*ρ*_*A*_ and *ρ*_*A*′_ are both independent of *φ*), indicating the necessity of entanglement in the input state for observing the photons’ wave-like feature. The dependences of *P*_00′_ = *P*_11′_ on *α* and *φ* for 0 < *q *< 1 and *θ *≠ *π*/4, are displayed in Fig. 4(a) and (b) for *q *= 1/2, while *θ * = *π*/8 and *θ * = 3*π*/8, respectively. Apart from the quantitative differences with respect to the imperfection-free situation (i.e., *q *= 1 and *θ *= *π*/4), the figures still qualitatively demonstrate the particle-like (wave-like) behavior at *α *= 0 (*α *= *π*/2) and a continuous transition between these two extreme cases as *α* gradually varies from 0 to *π*/2 or vice versa.

Next, we consider imperfection in the detection process. Namely, we assume that the photon detectors are not ideal, i.e., their efficiencies are smaller than 1. Let *η*_*m*_ < 1 (*η*_*m*′_ < 1) with *m *= 0, 1 be the efficiency of the imperfect detector *D*_*m*_ (*D*_*m*′_). Such a detector could be modeled by a perfect detector to be placed behind a lossless unbalanced beam-splitter with transmittivity *T*_*m*_ = *η*_*m*_ (*T*_*m*′_ = *η*_*m*′_) at which the photon of interest is superimposed with the vacuum. The transmitted part with probability *η*_*m*_ (*η*_*m*′_) propagates towards the perfect detector, while the reflected part with probability 1 − *η*_*m*_ (1 − *η*_*m*′_) is assumed to be absorbed by the environment. Then the collective detection probabilities *P*_*mm*′_ resulted from the imperfect detectors are proportional to 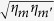. This though reduces the detection probabilities, but retains their dependences on *α* and *φ* unchanged for given detector efficiencies. In other words, one can still observe the continuous transition between the particle-like and wave-like behaviors of the two photons using inefficient detectors.

## Discussion

Following the successful investigation, both theoretical and experimental, of the quantum version of delayed-choice schemes for a single photon, we extend the idea to the case of two entangled photons. By sending each photon of the photon entangled pair to an interferometer and controlling the operation of Hadamard gate on one photon by an ancilla prepared in a quantum superposed state followed by measuring the ancilla in a suitable basis we can reveal the global properties of the two photons as particle-like, wave-like or both particle- and wave-like at the same time, though the independent behavior of an individual photon is always particle-like. By varying the ancilla state parameter we can also observe continuous transition from particle-like behavior to wave-like one and vice versa. We have proved that the obtained results cannot be a consequence of any HV theory which would yield inconsistence in the photons’ behaviors. An important contribution of the present work is proposal of a new scheme for observing two-photon wave-particle duality. Our scheme is efficient and experimentally feasible because it gets rid of the employment of ancilla, the controlling operation on the test system by the ancilla as well as the postselection conditioned on the necessary measurement on the ancilla. Therefore, in our scheme we can directly (i.e., without any postselection) observe the desired wave-particle superposed state of two photons as well as the continuous transition between the particle and wave behaviors of the two photons. Our scheme could be realized in the lab using current technologies.

## Additional Information

**How to cite this article:** Man, Z.-X. *et al*. Simultaneous observation of particle and wave behaviors of entangled photons. *Sci. Rep.* **7**, 42539; doi: 10.1038/srep42539 (2017).

**Publisher's note:** Springer Nature remains neutral with regard to jurisdictional claims in published maps and institutional affiliations.
